# Reinventing the Clinical Audit in a Pediatric Oncology Network

**DOI:** 10.1097/MPH.0000000000002591

**Published:** 2022-11-15

**Authors:** Carolyn Russo, Jennifer Morgan

**Affiliations:** * Department of Hematology; † Affiliate Program Office, St. Jude Children’s Research Hospital, Memphis, TN

**Keywords:** clinical audit, pediatric oncology, clinical networks

## Abstract

Providing equal access to pediatric cancer patients regardless of their geographic location is a major goal of the Affiliate Program at St. Jude Children’s Research Hospital (St. Jude). Thirty-five percent of new cancer patients enrolled on St. Jude clinical trials reside in the communities of 1 of the 8 affiliate clinics, which serve 9 states in the Southeast and Midwest United States. The affiliate clinics support participant recruitment for clinical trials and the geographic extension of St. Jude clinical care. To ensure high-quality pediatric cancer care, we instituted on-site clinical audits, however, we did not see improvement in clinical outcomes including the time to antibiotics in febrile immunocompromised patients, consistent hand-off communication, consistent documentation of oral chemotherapy, and adherence to a central line bundle in the ambulatory setting. We then moved to a more comprehensive clinical audit which involved self-reflection of clinic staff members, transparent data sharing, development of local quality champions, and engagement of senior leaders. The comprehensive approach was more successful in improving clinical outcomes including the time to antibiotics, hand-off communication, documentation of oral chemotherapy administration, and adherence to a central line bundle in the ambulatory setting.

Advancing cures for cancer requires clinical research trials. However, clinical research trials are only as informative as the participant enrollment. If the participants do not reflect the general population, it is difficult to generalize the results. One strategy to increase access for participants is to integrate clinical research trials in community health care systems.^1^ The Affiliate Program at St. Jude Children’s Research Hospital is an example of a team-based approach to engage community physicians in clinical research to benefit all children. To ensure high-quality care, we have developed a comprehensive clinical audit structure that can be applied in other cancer care hub-and-spoke networks.

The clinical audit can be an effective tool for improving health care, yet a Cochrane Collaboration review of 140 studies showed that clinical audits lead to only moderate improvement.^2^ Health care is personal. Many aspects of health care delivery are driven by behavior, and behavioral change is challenging. Thus, audit feedback alone may not produce desired results.^3^ We describe a comprehensive approach to improve the effectiveness of clinical audits measured by a decreasing number of audit findings after implementation. The approach included involving the clinic staff in self-reflection, transparent data sharing among the teams, development of local champions, engagement of senior leaders, and continuous monitoring of quality metrics.

## METHODS

**FIGURE 1:**
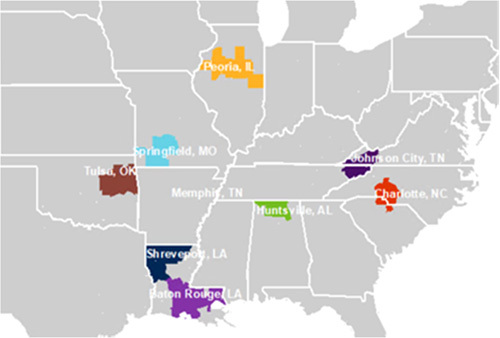
The 8 St. Jude Affiliate Clinics. The colored areas denote where 85% of the affiliate patients reside. St. Jude Children’s Research Hospital is in Memphis, Tennessee.

The goal of the St. Jude Affiliate Program is to allow more children to receive St. Jude care close to home and to increase access to pediatric oncology clinical research trials developed at St. Jude. The eight St. Jude affiliate clinics serve 9 states in the Southeast and Midwest (Fig. 1).

The affiliate institutions serve rural and suburban areas with a diverse demographic population. The affiliate clinics provide a substantial resource for patient recruitment for St. Jude clinical trials. During the years of this report, the affiliate clinics in aggregate saw an average of 302 new oncology patients per year of which an average of 38% of patients were enrolled on therapeutic primary clinical trials.

Each affiliate clinic is part of a not-for-profit health system. No affiliate clinic is part of an institution that provides pediatric stem cell transplantation, and none has pediatric hematology/oncology fellowship programs. Each clinic ranges in size and capacity. Four clinics had 40 to 60 new oncology patients per year and 4 clinics had 20 to 30 new oncology patients per year during the years of this report. The number of providers in each clinic ranged from 3 to 7. Ensuring high-quality care in smaller programs can be challenging and maintaining equitable high-quality care across a remote network is critical for patient safety and an optimal patient experience.

We instituted an on-site clinical audit to assess care in the affiliate clinics. Annually, an audit team composed of 1 physician and 1 nurse observed direct patient care in the clinic over a 2-day to 3-day period. They surveyed central line care, chemotherapy administration, patient teaching, blood product administration, and provider-patient interactions. Patient safety and confidentiality were maintained. The observers looked for adherence to a central line bundle based on national standards. During chemotherapy administration, the observers looked for independent dose calculations, verification of chemotherapy orders with the treatment schema, review of laboratory criteria, patient identification, and proper handling of cytotoxic therapy. Patient and family education, including anticipatory guidance, was reviewed. Thirty-six independent items were evaluated (Supplementary Table 1, Supplemental Digital Content 1, http://links.lww.com/JPHO/A584). After the first 2 years, the on-site clinical audit continued to demonstrate deficiencies without improvement. The most common deficiencies noted were inconsistent communication when patients transitioned between St. Jude and the affiliate clinics, delay in the time to antibiotics in febrile immunocompromised patients, inconsistent documentation of oral chemotherapy administration and the lack of adherence to a central line bundle in the ambulatory setting.

We then developed a more comprehensive approach to the clinical audit. First, we engaged the clinical team being audited. We began surveys of the clinic staff regarding perceptions of their clinic operations to identify perceived areas of strengths and weaknesses. This component enabled the clinic staff to indicate which aspects of their clinic were working well and which aspects could be improved, setting the tone for continuous quality improvement.

We identified provider champions for each clinic who received training in quality improvement using the American Society of Clinical Oncology Quality Training Program (ASCO QTP) (https://practice.asco.org/quality-improvement/quality-programs/quality-training-program). Each clinic had a dedicated nurse educator with protected time to lead projects. Joint quality improvement projects were facilitated by the Affiliate Nurse Director (J.M.), with multiple affiliate team members participating from various disciplines, including nursing, pharmacy, and physicians.

Each month, the affiliate clinic nurse educator submitted their local data on specific quality indicators to a secure dashboard. The dashboard tracked time to administration of antibiotics in immunocompromised children with fever, central line-associated bloodstream infections in ambulatory patients, patient/parent satisfaction scores, medication adverse events, and laboratory adverse events. We started sharing the individual quality metrics with the clinic team, and the data from each clinic was presented anonymously with team members of the 8 clinics.

The quality data from each affiliate clinic was shared annually with the respective hospital leadership, including chief medical officers, chief executive officers, and senior leaders.

## RESULTS

**FIGURE 2:**
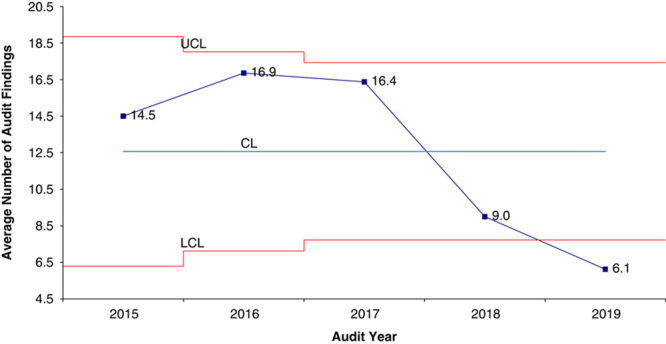
X-bar control chart of average number of audit findings per year from 2015 to 2019. The comprehensive clinical audit was implemented in 2017. CL indicates center line; LCL, lower control limit; UCL, upper control limit.

The clinical audit structure started as an annual, on-site audit. After the first 2 years 2 clinics showed minimal improvement, however, there was no improvement at the other 6 clinics. After instituting the additional audit components of self-reflection, data sharing, quality training, and engagement of senior leaders the number of findings at every clinic decreased (Fig. 2).

Building a team approach from the ground-up garnered more engagement with the clinical audit and the quality improvement efforts. The self-reflection surveys gave the clinic team members an opportunity to think about what was working and what needed improvement. The survey was informed by the most common deficiency noted at every clinic, which was inconsistent communication. The survey scored bidirectional communication and asked about barriers to high-quality care and suggestions for improvement. The response rate to the survey ranged from 36% to 38%. We shared the results with all the providers.

Rather than a directive being imposed upon them, the clinic staff took ownership of the audit findings, which in some cases led to the development of quality improvement projects. In one case, a clinic assumed the wait times in their clinic were adequate, but audit findings showed otherwise. This clinic initiated a quality improvement project to decrease wait times in the chemotherapy area.^4^

Transparent sharing of data with the teams provided opportunities for improvement. Quality metrics shared anonymously between the teams prompted some clinics to ask what higher-performing clinics were doing to improve outcomes. For example, when the central line-associated bloodstream infection rate in implanted catheters was shared between clinics, 1 clinic recognized a potential area for improvement. They learned from another clinic that infections were occurring when lines were accessed outside the pediatric oncology clinic (eg, emergency departments and diagnostic imaging suites). They instituted a teach-back method with parents of children with implanted catheters.^5^

Quality metrics were shared with the clinical teams and, importantly, with the hospital leadership of each institution. This step in the comprehensive approach was instrumental to ensure that the teams had resources to achieve the shared goals. For example, decreasing the time to administration of antibiotics in immunocompromised children with fever may not have been possible without senior leadership’s involvement. Because fever often develops in children at nights and on the weekends, when the clinics were closed, the outpatient clinic teams needed support from hospital leadership to ensure that prompt antibiotics administration occurred in emergency departments and on the inpatient hospital units.^5^

Training clinic team members as quality champions was critical to implement change. The champions received quality improvement training through the ASCO QTP. Having peers with proficiency in quality improvement increased the commitment to work through the audit findings. One finding noted in the clinical audits was inconsistent documentation of oral chemotherapy administration. With the coaches of the ASCO QTP, a team composed of members of 3 clinics performed a quality improvement project to improve compliance with oral chemotherapy documentation from a baseline of 17.4%. The team developed an aim statement, worked through a process map of the current state, created a cause-and-effect diagram, and studied 3 interventions. Compliance was improved to >85% within 6 months, and the team built a plan for sustainability (unpublished data).

## DISCUSSION

Integrating clinical research trials into the practice of community providers has been proposed as 1 mechanism to increase diversity of research participants.^1^,^6^ The St. Jude Affiliate Program is 1 example of this approach. However, ensuring high-quality care in the clinical network of remote sites can be challenging. We developed a comprehensive clinical audit process that decreased deficiencies noted in the clinical audits after implementation.

As described in the literature, the effectiveness of audits is not uniformly positive.^7^ The nature of an external reviewer giving feedback may appear dictatorial rather than engaging and collaborative. Our initial experience was similar. We found that a simple, yearly clinical audit did not yield continuous improvement.

Adding a team-based approach involving all stakeholders was more successful. Because each individual component was not independently evaluated, we do not know the benefit of each component; nonetheless, the comprehensive approach was engaging and provided more sustainable quality improvement.

Starting with a bottom-up process, set the tone for a more-inclusive approach to quality improvement. As noted by others, self-reflection is a method to garner engagement with the clinical audit.^7^

The sharing of data was key. Rather than the team members making assumptions about how the clinic was operating, examining the data objectively showed how the clinic was functioning. Transparent data sharing can be motivating and reinforces the team approach.^8^,^9^ Sharing the data with hospital leadership is a tool to help get support for resources when necessary.

The training in quality improvement equipped the teams with knowledge, skills, and attitudes to champion culture change in their clinical practices. Successful quality improvement works when clinicians lead; however, they must know how to design, implement, and evaluate projects. The ASCO QTP provided the tools to practice continuous quality improvement (https://practice.asco.org/quality-improvement/quality-programs/quality-training-program).

Using a comprehensive approach that involves self-reflection, transparency of data sharing, development of local champions, and engagement of senior leaders, we have been successful in quality improvement across a broad geographic pediatric oncology network. This strategy may be used with other clinical networks working to increase access to clinical research trials in community health care systems.

## Supplementary Material
