# Supplementary material for: Reinventing the Clinical Audit in a Pediatric Oncology Network
Source: J Pediatr Hematol Oncol. 2022 Nov 15;45(4):e483–6. doi: 10.1097/MPH.0000000000002591 (PMC10115487; doi:10.1097/MPH.0000000000002591)
Supplement: Supplementary file 1 [file mph-45-e483-s001.docx]

**Supplemetnal Digital Content 1.**

| Age at diagnosis of NF1 (years) | Form of NF1 | Genetic testing available? | Sex | Age at tumor diagnosis (years) | Time from symptoms to treatment (months) | Nutritional support | Visual impairment | 1^st^ line therapy | response to treatment | 2^nd^ line therapy |
| --- | --- | --- | --- | --- | --- | --- | --- | --- | --- | --- |
| 0.25 | familial | no | f | 2.3 | 6 | ng tube | no | vbl/bevacizumab | response | none |
| 0.5 | familial | no | f | 7.2 | 7.1 | nutrition counseling, high caloric drinks | no | vbl | response | none |
| 0.9 | sporadic | yes (heterozygous mutation in NF1 gene at c.3942G>A) | f | 2.1 | 24.2 | g-tube | no | vbl | partial response, progression after end of treatment | trametinib |

nf1 = neurofibromatosis type 1; f = female; vbl = vinblastine; ng tube = nasogastric tube; g-tube = gastrostomy tube

**Supplemetnal Digital Content 1.**

| Reference | number of patients (gender) (n=14) | age (years)  (median 1.77, range 0.5-5.8) | initial presentation | treatment | response to treatment/clinical course |
| --- | --- | --- | --- | --- | --- |
| Cavicchiolo, M.E., et al., Diencephalic syndrome as sign of tumor progression in a child with neurofibromatosis type 1 and optic pathway glioma: a case report. Childs Nerv Syst, 2013. 29(10): p. 1941-5. | 1 (male) | 3 | 2-month history of progressive weight loss (-2.5 kg), emaciation and poor feeding  (12 months before diagnosis, OPG was already diagnosed radiologically) | carboplatin and vincristine, regimen changed because of allergy to carboplatin | weight increase (normalized) after 6 months of treatment, tumor size remained stable under treatment, no longer follow-up data available |
| De Martino, L., et al., Diencephalic Syndrome Due to Optic Pathway Gliomas in Pediatric Patients: An Italian Multicenter Study. Diagnostics (Basel), 2022. 12(3). | 4  (2female,  2 male) | median 1.77 (range 1.17-2.17) | BMI below -2 SDS with additional visual symptoms | upfront chemotherapy (3 patients according to SIOP LGG 2004, 1 according to HIT-LGG 1996) regimens  none received radiotherapy | partial response or stable disease with increasing weight in all patients  all 4 showed progression (between 1 and 4 progressions over time, no information on weight loss)  followed for a median time of 8.2 years (range 1-15.2 years) |
| Kilday, J.P., et al., Favorable survival and metabolic outcome for children with diencephalic syndrome using a radiation-sparing approach. J Neurooncol, 2014. 116(1): p. 195-204. | 2  (1female, 1 male) | 2.14 (female) and 2.67 (male) | weight/height percentile <2^nd^ or 2^nd^-5^th^ percentile,  additional emesis and optic atrophy (female) and additional developmental delay (male) | treated with carboplatin and vincristine, changed to vinblastine in both patients because of allergy to carboplatin | increasing weight after 6 months, normalization after 12 months  girl without progression (followed for 12.7 years)  boy with 4 progressions and further therapy with surgery (PR), thioguanine/vincristine/CCNU, irinotecan and bevacizumab  (followed for 8.4 years) |
| Pilotto, C., et al., Diencephalic syndrome in child with NF-1 and hypothalamic tumour. 2018. 2. | 1 (male) | 1.67 | 5 months history of weight loss (50^th^-2^nd^ centile), vomiting, poor feeding, rapid head growth and developmental regression | carboplatin and vincristine (one cycle), after worsening symptoms debulking and VP-shunt, post surgery  treatment changed to vinblastine | improvement of symptoms (increasing weight, improved vision, development) with reduction in tumor size |
| Rakotonjanahary, J., et al., Mortality in Children with Optic Pathway Glioma Treated with Up-Front BB-SFOP Chemotherapy. PLoS One, 2015. 10(6): p. e0127676. | 2 (female) | 0.5 and 0.9 | diencephalic syndrome (not further specified) with intracranial hypertension (0.5 year-old); no further information on the 0.9 year-old. | upfront chemotherapy according to BB-SFOP (carboplatin, etoposide, cisplatin, vincristine and cyclophosphamide)  older patient received radiation at progression | younger patient died because of chemotherapy induced complication (0.3 years after diagnosis)  older patient died 17 years after diagnosis  (tumor progression) |
| Santoro, C., et al., Pretreatment Endocrine Disorders Due to Optic Pathway Gliomas in Pediatric Neurofibromatosis Type 1: Multicenter Study. J Clin Endocrinol Metab, 2020. 105(6). | 4 (female) | median age of 4.66 (1.4-5.8) | no further symptoms mentioned besides diencephalic syndrome symptom complex, not described in detail | all patients received chemotherapy (regimen not mentioned) |  |

BMI = body mass index; OPG = optic pathway glioma; PR = partial resection; SDS = standard deviation score; VP = ventriculoperitoneal
